# Sociodemographic and Clinical Determinants of Adults With Advanced HIV Disease on Antiretroviral Therapy in West Oromia, Ethiopia: A Hospital‐Based Retrospective Study

**DOI:** 10.1155/arat/6666071

**Published:** 2026-05-25

**Authors:** Geleta Asebe Hinkosa, Sheillah Hlamalani Mboweni

**Affiliations:** ^1^ University of South Africa, Department of Health Studies, College of Human Sciences, P. O. Box 392 Preller Street Muckleneuk, Pretoria, South Africa, unisa.ac.za

**Keywords:** adults living with HIV, advanced HIV disease, antiretroviral therapy, determinants associated with advanced

## Abstract

**Background:**

HIV/AIDS remains a significant public health challenge, with many individuals developing advanced HIV disease despite the scale‐up of antiretroviral therapy worldwide. Determinants related to sociodemographics, clinical practice and laboratory service associated with advanced HIV disease are not well known in sub‐Saharan Africa, such as Ethiopia. This study examines the potential sociodemographics and clinical practice of determinants of advanced HIV disease among adults on antiretroviral therapy in the West Oromia Region, Ethiopia.

**Methods:**

A hospital‐based retrospective study was employed. The Raosoft online sample calculator was used to estimate a sample of 544 patient medical records in the study. A systematic sampling technique was used to select participants who met the inclusion criteria from three purposively selected public hospitals between January 2017 and December 2021. Descriptive statistics were applied to the dataset after it was cleaned and validated in EpiData, and the final analysis was performed in SPSS Version 29. Bivariate and multivariate logistic regression models were used to analyse sociodemographic and clinical practice factors associated with advanced HIV disease, with *p* < 0.05.

**Results:**

The study revealed sociodemographic and clinical practice determinants associated with advanced HIV disease among adults, which includes male sex (adjusted odds ratio [AOR] = 1.85 and 95% confidence interval [CI] 1.18–2.90), the presence of HIV signs and symptoms (AOR = 2.83 [95% CI 1.74–4.61]) and OIs (AOR = 3.44 [95% CI 2.01–5.92]) at the time of antiretroviral therapy enrolment, bedridden and ambulatory health conditions (AOR = 3.58 [95% CI 1.76–7.31]), and low CD4 cell count of less than 200 cells/mm3 accounted for 127 (36.5%) at treatment initiation while having CD4 test results at baseline data collections participants (AOR = 1.97 [95% CI 1.20–3.23]), and 84 (37%) of those delayed ART initiation (AOR = 1.75 [95% CI 1.08–2.83]). 42 (7.7%) had ART on ≤ 1–7 days and 143 (26.3%) in > 7 days. These significantly correlated with the development of advanced HIV disease or OIs at a *p* value of < 0.05 among this study population. Only 359 (66%) had same‐day ART.

**Conclusion:**

The findings highlight the importance of early HIV diagnosis and initiation of antiretroviral therapy, adherence to treatment regimens and monitoring for OIs in the prevention and management of advanced HIV disease. Therefore, the study calls for male‐targeted interventions, strengthening the capacity of healthcare providers, and a review of protocols for early HIV diagnosis and care to enhance patient health outcomes.

## 1. Introduction

Advanced human immunodeficiency virus (HIV) disease (AHD) remains a critical global health issue for people living with HIV (PLHIV) [1, 2]. AHD is defined as a CD4 count below 200 cells/mm^3^ or the presence of severe symptoms marking World Health Organisation (WHO) Stages III or IV [3]. This is concerning for adults on antiretroviral therapy (ART). Despite increased ART access in over 135 countries [4], opportunistic infections (OIs) like tuberculosis (TB) and cryptococcal meningitis (CM) remain leading causes of death among AHD patients [5]. Although more healthcare providers (HCPs) are trained on HIV management, more frequent clinical and programme monitoring is required [6, 7]. In 2023, 38.6 million people lived with HIV, causing 560,000 deaths, surpassing the 2025 target [8]. AHD presentations in low‐ and middle‐income countries rose from 14.5% to 71% from 2012 to 2016 [9–13]. Understanding the determinants of AHD is essential for reducing mortality in PLHIV and improving health outcomes.

Most studies in sub‐Saharan Africa indicate that sociodemographic factors—such as older age, male sex, low education, marital status and heterosexual transmission—are linked to a higher risk of AHD compared to non‐AHD patients [14–17]. However, a study in Rwanda found no significant differences in sex or distance to health facilities between AHD and non‐advanced HIV patients [18]. This warrants further investigation in Africa, particularly in Ethiopia. Additionally, rural residents are also at greater risk than urban dwellers [19]. Identifying these factors is crucial for improving HIV treatment and reducing transmission.

Clinical characteristics, including advanced clinical staging and low haemoglobin levels, are associated with a higher likelihood of presenting with AHD. Patient‐related factors like low‐risk perception and lack of awareness of HIV symptoms contribute to higher AHD rates [20, 21]. Furthermore, a cross‐sectional study conducted between 2008 and 2020 in China reports that heterosexual transmission and diagnosis at sexually transmitted infection clinics and hospitals are associated with increased risk of AHD at presentation [16]. Clinical services, patient‐related factors and laboratory investigations were not addressed, which means the implementation of a package of care for AHD is low. Conducting a study to identify such factors may play a great role in successful HIV care and treatment and reduction in HIV transmission.

Ethiopia faces significant missed opportunities in screening, diagnosing and treating OIs in public hospitals. The country also has a high prevalence of AHD, with 60% of adults starting ART already in this stage [22]. This raises concerns about compliance with healthcare policies. A study found that AHD affects more men (66%) than women (56%), particularly in rural areas, necessitating further investigation [22]. Additionally, 16.9% of men and 26.5% of women aged 15 to 64 had a CD4 count below 200 cells/mm3, indicating a 22.0% occurrence of AHD [23]. However, no studies have explored AHD factors in this study area. Hence, this study aims to identify sociodemographic and clinical practice risk factors for AHD among adults on ART to develop targeted interventions to improve health outcomes for PLHIV in West Oromia, Ethiopia.

## 2. Methods

### 2.1. Study Setting

The study was conducted at Ambo General Hospital, Nekemte Comprehensive Specialised Hospital and Shambu General Hospital, located in Ambo Town, Nekemte Town and Shambu Town, respectively, in the western part of Oromia, Ethiopia. These facilities were selected purposefully because they had CD4 machines used to determine factors associated with AHD and had experienced HCPs for providing baseline and follow‐up HIV care and treatment services for the newly started ART. For each patient who started on ART, all demographics and healthcare delivery system information are recorded in the facility’s paper‐based registers and kept in the electronic medical records (EMRs). Adults who had started on ART from January 2017 to December 2021 were included in the study area.

### 2.2. Study Design and Population of the Study

A hospital‐based retrospective study was conducted to identify determinants of AHD among adults on ART. This design collects secondary data from 2017 to 2021 from a sample, allowing researchers to explore factors associated with AHD that could be solved to improve health outcomes for adults living with HIV (ALHIV).

### 2.3. Inclusion and Exclusion Criteria of the Study

The study population included ALHIV over 18 who had started ART between January 2017 and December 2021 in the three selected public hospitals, with their clinical and psychosocial support related to HIV care being documented in their medical records, including baseline findings of CD4 cell count and WHO Stages I, II, III and IV being recorded for inclusion in this study review. Moreover, the date of HIV diagnosis and ART initiation should also be recorded.

Exclusions were made for those under 18, patients without complete medical records, pregnant women and those transferred from other facilities.

### 2.4. Sample Size Determination

The sample size was calculated using the Roasoft online estimation formula, assuming a 95% confidence level, 5% margin of error, and an expected AHD prevalence of 40.1% from previous research [21]. A total sample size of 363 was calculated from 1322 medical records, retrieved from the EMR ART system, with an additional 15% added for missing data, resulting in 544 adults included, with proportional allocations among the three selected hospitals.

### 2.5. Sampling Methods

A systematic sampling technique was used to select adults who started ART between 2017 and 2021 to identify determinants of AHD. This method is efficient, reduces costs, saves time in data extraction, and enhances population representativeness [24]. Each ALHIV is assigned a unique ART number (UAN) upon diagnosis. The UAN is a standardised number given to all PLHIV in the facilities based on the guidelines of Ethiopia. A list of 1322 medical records was retrieved from the EMR ART system and was part of the sampling frame. Then, by using the every second records interval of the sample frame, a total of 544 study participants were selected from medical records in the three selected public hospitals in western Oromia.

### 2.6. Data Collection

A retrospective chart review using a structured checklist was used to identify the determinants associated with AHD among adults initiated on ART. A structured English language checklist was used, adapted from the Ethiopia Demographic and Health Survey (EDHS) 2016 and WHO ART guidelines [25]. Factors related to sociodemographics, clinical practice and laboratory characteristics were extracted.

Two data collection supervisors with MPH degrees and three trained data collectors with BSc in nursing and at least 2 years of clinical experience conducted the data collection. Each data collector worked with a supervisor to ensure accuracy by reviewing the inclusion criteria and forms. Data collection occurred from February to April 2024, primarily reviewing ART registers, follow‐up forms, and laboratory investigation forms to assess baseline demographics and healthcare provisions.

### 2.7. Data Management and Analysis

Data collectors received 3 days of training on extracting data from medical records. During data collection, personally identifiable information was omitted, using numbers instead of names to ensure confidentiality. Supervisors checked data completeness and consistency daily. After data collection, feedback was provided to maintain study consistency, and data completeness was verified using Microsoft Excel checklists.

EpiData Version 4.6.0.6 and SPSS Version 29 were used for data entry and analysis. Characteristics of AHD were presented in tables and figures, showing means, percentages and medians. Odds ratios were calculated, with a *p* value of less than 0.05 considered statistically significant [26]. Missed values like viral load determination at 6 months (45.6%), viral load measurement at 12 months (75.9%) and mental health assessment at enrolment (16.5%) were deleted from data analysis. Logistic regression models assessed associations between AHD and various characteristics. Model fitness was evaluated using the Hosmer–Lemeshow test, and results were reported through text, tables and graphs.

### 2.8. Validity and Reliability to Ensure the Rigour of Quantitative Studies

Validity pertains to the accuracy of scientific findings regarding inferences of the effect of independent variables on dependent variables [27]. Reliability refers to the consistency and repeatability of information obtained [28, 29]. In this study, validity was established by using experienced nurses for data collection, ensuring accurate and consistent data. A structured checklist was pretested in a nearby public hospital to confirm its effectiveness. Data were sourced from reliable existing records, with measures in place to ensure stability and consistency throughout the process.

### 2.9. Ethical Considerations

Ethical clearance was obtained from the University of South Africa’s College of Human Sciences Research Ethics Committee (CREC) on 23 November 2023, reference number: 19946546_CREC_CHS_2023, and the Oromia Health Bureau on 21 November 2023, BFO/UBTFU/1‐16/912. Permission to access patient medical records was granted by hospital managers. To ensure confidentiality, personally identifiable information, patients and hospital names were excluded during data extraction.

## 3. Results

### 3.1. Sociodemographic Characteristics of Participants

The study included 544 adults initiated on ART, analysing their sociodemographic characteristics presented in Table 1. Females made up a higher proportion, with 307 participants (56.4%), compared to 237 males (43.6%). The majority were aged 35–44 years (199 participants, 36.6%), with a mean age of 35.5 years. Most participants lived in urban areas (358, 65.8%), while 186 (34.2%) resided in rural areas. The predominant religion was Protestant (257, 47.2%), followed closely by Orthodox Christians (250, 46%), with Muslims (29, 5.3%), Catholics (4, 0.7%), and others (4, 0.7%) comprising smaller percentages. In terms of education, 192 participants (35.3%) had primary education, while 138 (25.4%) had secondary and 67 (12.3%) had tertiary education; 147 (27%) were uneducated. Most participants were married (345, 63.4%), with 177 (32.5%) unemployed, while others included farmers (107, 19.7%), daily labourers (72, 13.2%), government employees (66, 12.1%) and merchants (53, 9.7%).

**TABLE 1:** Sociodemographic characteristics of participants (*n* = 544) (source: researchers’ own work).

Characteristics	Frequency	Percent (%)
*Age (n = 544)*		
18–24	53	9.7
25–34	184	33.8
35–44	199	36.6
45–54	83	15.3
≥ 55	25	4.6

*Sex (n = 544)*		
Female	307	56.4
Male	237	43.6

*Residence (n = 544)*		
Urban	358	65.8
Rural	186	34.2

*Religion (n = 544)*		
Protestant	257	47.2
Orthodox Christian	250	46
Muslim	29	5.3
Catholic	4	0.7
Others	4	0.7

*Education level (n = 544)*		
No education	147	27
Primary education	192	35.3
Secondary education	138	25.4
Tertiary education	67	12.3

*Marital status (n = 544)*		
Single	65	11.9
Married	345	63.4
Divorced	85	15.6
Widowed	49	9.1

*Occupation status (n = 544)*		
Unemployed	177	32.5
Farmer	107	19.7
Daily labourer	72	13.2
Government employees	66	12.1
Merchants	53	9.7
Drivers	14	2.6
Nongovernment employees	4	0.7
Others	51	9.4

### 3.2. Clinical Practice and Laboratory Testing Characteristics of Participants

As shown in Table 2, most adults initiated on ART (333, 61.2%) had no signs or symptoms of HIV/AIDS at baseline, while 211 (38.8%) did. Among those with signs and symptoms of HIV, chronic cough for over a month (86, 40.8%) and weight loss (69, 32.7%) were the most common at enrolment in HIV care. A total of 378 participants (69.5%) presented without OIs, while 166 (30.5%) had one or more OIs. Among the 166 participants (30.5%) who presented with OIs, predominantly TB was the most frequently reported OI, affecting 70 (42.2%) participants. This was followed by oral candidiasis, 17 (10.2%), herpes zoster, 11 (6.6%), CM, 6 (3.6%), and severe bacterial infections (SBIs), 6 (3.6%). Other OIs were documented in 56 (33.7%) participants. Additionally, 3 (1.8%) participants were reported as having two or more of these types of OI findings at baseline. Screening indicated that 525 participants (96.5%) were screened for TB among participants of the study.

**TABLE 2:** Clinical practice and laboratory testing characteristics of participants (*n* = 544) (source: researchers’ own work).

Characteristics		Frequency	Percent (%)
Had signs and symptoms at baseline			
Yes		211	38.80
No		333	61.20

*Presence of OIs at enrolment*			
Yes		166	30.5
No		378	69.5

Types of OIs (*N* = 166)	TB		
	Yes	70	42.2
	No	96	57.8
	Oral candidiasis		
	Yes	17	10.2
	No	149	89.8
	Herpes zoster		
	Yes	11	6.6
	No	155	93.4
	Cryptococcal meningitis		
	Yes	6	3.6
	No	160	96.4
	Several bacterial infections		
	Yes	6	3.6
	No	160	96.4
	Others		
	Yes	56	33.7
	No	107	66.3

*TB screened at baseline*			
Yes		525	96.5
No		19	3.5

*BMI status at enrolment*			
≤ 18.49 kg/m2		161	29.6
18.50–24.99 kg/m2		377	69.3
≥ 25 kg/m2		6	1.1

*Functional status at enrolment*			
Working		462	84.9
Ambulatory		62	11.4
Bedridden		20	3.7

*Haemoglobin testing at enrolment*			
Yes		205	37.7
No		339	62.3

*Haemoglobin level (N = 205)*			
≤ 11 gm/dL		41	20
≥ 11.01 gm/dL		164	80

*CD4 cell count testing (N = 544)*			
Yes		348	64
No		196	36

*CD4 cell count results (N = 348)*			
≤ 200 cells/mm3		127	36.5
≥ 200.01 cells/mm3		221	63.5

*WHO staging (N-544)*			
I/II		370	68
III/IV		174	32

*ART initiation (N = 544)*			
The same day ART initiation		359	66
1–7 days ART initiation		42	7.7
> 7 days ART initiation		143	26.3

*Viral load determination at 6 months (N = 249)*			
< 50 copies/mL		222	89.2
51–1000 copies/mL		16	6.4
> 1000 copies/mL		11	4.4

*Viral load determination at 12 months (N = 413)*			
< 50 copies/mL		395	95.6
51–1000 copies/mL		10	2.4
> 1000 copies/mL		8	2

*Mental health assessment at enrolment (N = 480)*			
Yes		90	18.7
No		390	81.3

*HIV testing approach*			
VCT		126	23.2
PITC		418	76.8

*Provision of TPT prophylaxis at enrolment*			
Yes		376	69.1
No		168	30.9

*Provision of CPT prophylaxis at enrolment*			
Yes		443	81.4
No		101	18.6

*Linkage to social support*			
Yes		164	30.1
No		380	69.9

*Presence of < 15 children in the family*			
Yes		402	73.9
No		142	26.1

Functional status revealed that 462 participants (84.9%) were functional, while 62 (11.4%) were ambulatory, and 20 (3.7%) were bedridden. Heamoglobin (HB) tests were performed on 205 participants (37.7%), with 164 (80%) having levels above 11 g/dL, indicating that 41 (20%) had aneamia.

Of the 544 participants included in the study, 348 (64.0%) underwent CD4 cell count testing at the time of ART initiation through the central laboratory platform, while 196 (36.0%) did not have a CD4 test performed at baseline. Among those tested, the largest proportion, 221 (63.5%), had CD4 cell counts greater than 350 cells/mm^3^, indicating relatively preserved immune function and earlier‐stage HIV disease. Notably, 127 (36.5%) participants had CD4 cell counts below 200 cells/mm^3^, meeting the clinical definition of AHD and reflecting significant immunosuppression at enrolment into HIV care and treatment.

A total of 401 participants (73.7%) were initiated on ART early, with 359 (66.0%) starting treatment on the same day or within seven days of HIV diagnosis, in line with WHO recommendations. In contrast, 143 (26.3%) initiated ART late, indicating delays in treatment initiation either by patients or HCPs. The largest proportion of participants, 370 (68%), were classified as WHO Stage I and II at enrolment into HIV care and treatment, while a smaller proportion, 174 (32%), were classified as WHO Stage III and IV at baseline. Viral load determination at 6 months, 222 (89.2%), resulted in < 50 copies, while others had values greater than 50 copies/mL. Viral load measurement at 12 months 395 (95.6%) resulted in < 50 copies/mL, while others > 50 copies/mL. Mental health assessment at enrolment was assessed for 90 (18.7%), while others were not assessed for participants enrolled in the study.

The HIV testing approaches used by most participants in the study, 417 (76.8%), knew their HIV status through provider‐initiated testing and counselling (PITC), compared to 126 (23.2%) individuals who knew their HIV status through voluntary counselling and testing (VCT). Most participants, 376 (69.1%), were started on tuberculosis preventive therapy (TPT), though 168 (30.9%) were not receiving the provision of TPT services, and this also raises concern as to why HCPs missed these patients for screening or they might be having signs of TB. In addition, even though the majority of participants, 443 (81.3%), have received the provision of cotrimoxazole preventive therapy (CPT) prophylaxis, some were not provided CPT prophylaxis, 101 (18.6%), at baseline data collection. In this study, poor implementation of guidelines and protocols by HCPs raises concern and should be addressed.

Most participants, 380 (69.9%), did not get a service linkage to social support, while only 164 (30.1%) were linked to social support services to different organisations and communities at the beginning of ART services. Lack of support services raises concern as it may contribute to poor adherence to treatment. Four hundred and two (73.9%) participants had children who were less than 15 years old in their families, and 142 (26.1%) of the participants had no children who were less than 15 years of age. The children less than 15 years living with ALHIV should be provided with HIV index contact testing for early diagnosis and treatment. Because the patient records had specifically asked about the children’s status, which requires encouragement for HCPs.

### 3.3. Statistical Analysis of Factors Associated With AHD

In the bivariate analysis of factors associated with AHD, 14 variables with *p* ≤ 0.25 were identified for multivariate logistic regression. These included age at diagnosis, sex, presence of signs and symptoms, OIs, BMI, functional status, HB testing, CD4 count testing, HIV testing approach, isoniazid preventive therapy (IPT), cotrimoxazole prophylaxis therapy (CPT), delayed ART initiation, social support, and presence of children under 15 in the family as candidates for the multivariate logistic regression analysis, as summarised in Table 3.

**TABLE 3:** Bivariate and multivariate analysis of factors associated with AHD in West Oromia, Ethiopia (*p* value < 0.05) (source: researchers’ own work).

Variables	Category	Total *n* (%)	AHD *n* (%)	Non‐AHD *n* (%)	Corollary (95% CI)	AOR (95% CI)	*p* value
Age	18–24 years	53 (9.7)	20 (37.8)	33 (62.2)	1	1	
	25–34 years	184 (33.8)	61 (33.2)	123 (66.8)	0.82 (0.43.1.54)	0.80 (0.36.1.78)	0.59
	35–44 years	199 (36.6)	88 (44.2)	111 (55.8)	1.31 (0.70.2.44)	1.47 (0.67.3.26)	0.341
	45–54 years	83 (15.3)	50 (60.2)	33 (39.8)	2.50 (1.23.5.08)	1.83 (0.74.4.49)	0.189
	≥ 55 years	25 (4.6)	8 (32.0)	17 (68.0)	0.78 (0.28.2.13)	0.55 (0.16.1.88)	0.340

Sex	Female	307 (56.4)	110 (35.8)	197 (64.2)	1	1	
	Male	237 (43.6)	117 (49.4)	120 (50.6)	1.75 (1.24.2.47)	1.85 (1.18.2.90)	0.007**^∗∗^**

Presence of signs and symptoms of HIV/AIDS	Yes	211 (38.8)	143 (67.8)	68 (32.2)	6.23 (4.26.9.11)	2.83 (1.74.4.61)	**<** **0.001** ^**∗∗∗**^
	No	333 (61.2)	84 (25.2)	249 (74.8)	1	1	

Presence of OIs at enrolment	Yes	166 (30.5)	127 (76.5)	39 (23.5)	9.05 (5.92.13.8)	3.44 (2.01.5.92)	**<** **0.001** ^**∗∗∗**^
	No	378 (69.5)	100 (26.5)	278 (73.5)	1	1	

BMI status at baseline	≤ 18.49 kg/m^2^	161 (29.6)	97 (60.2)	64 (39.8)	2.74 (1.87.3.99)	1.60 (0.97.2.64)	< 0.066
	≥ 18.50 kg/m^2^	383 (70.4)	130 (33.9)	253 (66.1)	1	1	

Functional status at enrolment	Working	462 (84.9)	164 (35.5)	298 (64.5)	1	1	
	Ambulatory and bedridden	82 (15.1)	63 (76.8)	19 (23.2)	6.03 (3.49.10.4)	3.58 (1.76.7.31)	**<** **0.001** ^**∗∗**^

Haemoglobin testing at enrolment	Yes	205 (37.7)	103 (50.2)	102 (49.8)	1.75 (1.23.2.45)	1.49 (0.94.2.36)	0.093
	No	339 (62.3)	124 (36.6)	215 (63.4)	1	1	

CD4 cell count test at enrolment (cells/mm3)	Yes	348 (64.0)	163 (46.8)	185 (53.2)	1.82 (1.26.2.62)	1.97 (1.20.3.23)	0.007**^∗∗^**
	No	196 (36.0)	64 (32.7)	132 (67.3)	1	1	

ART initiation	Early ART	401 (73.7)	143 (35.7)	258 (64.3)	1	1	
	Late ART	143 (26.3)	84 (58.7)	59 (41.3)	2.57 (1.74.3.80)	1.75 (1.08.2.83)	0.022**^∗^**

HIV testing approach	VCT	126 (23.2)	46 (36.5)	80 (63.5)	1	1	
	PITC	418 (76.8)	181 (43.3)	237 (56.7)	1.33 (0.88.2.00)	1.15 (0.69.1.92)	0.602

Provision of IPT prophylaxis at enrolment	Yes	376 (69.1)	128 (34.0)	248 (66.0)	0.36 (0.25.0.52)	0.61 (0.36.1.02)	0.060
	No	168 (30.9)	99 (58.9)	69 (41.1)	1	1	

Provision of CPT prophylaxis at enrolment	Yes	443 (81.4)	204 (46.0)	239 (54.0)	2.89 (1.75.4.78)	1.70 (0.91.3.19)	0.096
	No	101 (18.6)	23 (22.8)	78 (77.2)	1	1	

Linkage of social support at enrolment	Yes	164 (30.1)	62 (37.8)	102 (62.2)	0.79 (0.54.1.15)	1.05 (0.65.1.68)	0.855
	No	380 (69.9)	165 (43.4)	215 (56.6)	1	1	

Presence of children < 15 in the family	Yes	402 (73.9)	158 (39.3)	244 (60.7)	1	1	
	No	142 (26.1)	69 (48.6)	73 (51.4)	1.46 (0.99.2.15)	1.26 (0.78.2.07)	0.361

*Note:* Bold values are given in Table for variables significantly associated with the dependent variable (AHD) in the multivariate analysis. This is important for differentiating variables that are statistically significant from those not associated with AHD in multivariate analysis.

^∗^Significantly associated with AHD (*p* < 0.05).

^∗∗^Very significantly associated with AHD (*p* < 0.01).

^∗∗∗^Highly significantly associated with AHD (*p* < 0.001).

The multivariate analysis revealed six significant factors associated with AHD (*p* < 0.05): being male (adjusted odds ratio [AOR] 1.85), presence of signs and symptoms (AOR 2.83), presence of OIs (AOR 3.44), having a CD4 test (AOR 1.97), abnormal functional status (AOR 3.58), and delayed ART initiation (AOR 1.75). The goodness of fit of the model was assessed using a Hosmer and Lemeshow test, yielding a *p* value of 0.648.

## 4. Discussion

The study identified sociodemographic and clinical practice factors significantly associated with AHD, including sex, where males have a higher prevalence of AHD than females, the presence of signs and symptoms of HIV and OIs, bedridden functional status clients, having CD4 test results, and late initiation of ART from the participants’ medical records.

### 4.1. Sex and Its Association With AHD

The study found that AHD is more prevalent among males initiated on ART, with males being 1.85 times more likely to have AHD than females. This aligns with studies in sub‐Saharan Africa, Uganda and Tanzania, indicating that male gender is a significant risk factor for AHD [10, 14, 21]. Contributing factors include late diagnosis, stigma and lower engagement in care. Among participants, 71 males (55.9%) had lower CD4 counts, which may hinder recovery, and 193 males (44.9%) had lower disclosure rates compared to females in this study. This suggests the need for gender‐targeted interventions to improve early presentation, CD4 testing, retention and disclosure of HIV status.

### 4.2. Clinical Practice Factors as a Determinant of AHD

The study identified clinical practices as predictors of AHD, including the presence of signs and symptoms of HIV and OIs, having CD4 cell count results, bedridden and ambulatory functional status and delayed ART initiation in patients. Patients often seek care late, presenting signs and symptoms with cough (40.8%), weight loss (32.7%), diarrhoea (10.4%) and OIs, which are significantly associated with AHD. These findings are consistent with studies in China and Sierra Leone, which reported higher rates of respiratory infections and diarrhoea in AHD patients. Additionally, males exhibited a higher prevalence of HIV signs and symptoms [21–23]. This highlights the need to improve community awareness about regular HIV testing and timely medical care to prevent AHD.

In this study, TB (13.3%) and CM (3.6%) were the most common OIs among adults with AHD. This aligns with findings from several African countries, including Lesotho, South Africa, Mozambique, Kenya, Cameroon and Tanzania, which reported a higher prevalence of TB in AHD patients [30–37]. Contributing factors may include lack of education, alcohol consumption, low income and inadequate AHD care. Other studies in Uganda (4.4%), Ghana (0.6%), Vietnam (3.1%) and Ethiopia (3.8%) reported higher incidences of CM, attributed to poor compliance with protocols, inadequate screening and shortages of cryptococcal antigen reagents [38–42]. The study raised concerns about the provision of tuberculosis preventive treatment (TPT), fluconazole pre‐emptive therapy (FPT) and CPT for PLHIV, as inadequate treatment could lead to increased morbidity and mortality. Community education and early health‐seeking behaviours are crucial for improving patient outcomes. Additionally, malnutrition affected 29.6% of PLHIV, consistent with findings from Nigeria that linked it to poor nutritional screening and limited ART access [43]. Enhancing nutritional education and BMI assessments at enrolment can help identify malnutrition early.

The study also found that participants with CD4 test results were nearly twice as likely to be at risk for AHD, while 196 (36%) did not undergo CD4 testing at ART enrolment, which might have been missed for early identification of AHD. This underscores the need for better adherence to guidelines among healthcare professionals. Similar issues were noted in Iran, Zambia and Malawi, where high percentages of participants lacked CD4 tests due to malfunctioning equipment and unfamiliarity with testing processes [20, 44]. Implementing strategies to improve CD4 testing and ensure the necessary supplies are essential.

The study found that ALHIV in a severe bedridden state and ambulatory are four times more likely to have AHD compared to those who can perform their daily activities. This aligns with research from southeast Tanzania and Ethiopia, which highlighted the link between impaired functional status and AHD, often due to delays in seeking care and a shortage of trained HCP3s [30, 40]. Hence, facility‐based targeted interventions are needed to improve the functional status of the clients.

Additionally, participants who started ART late were nearly twice as likely to have AHD compared to those who initiated treatment early, a finding supported by studies from East Africa and Nigeria. This underscores the importance of increasing access to ART and ensuring timely initiation to reduce AHD. Factors contributing to late ART initiation include severe OIs, inadequate health services, behavioural perceptions, lack of HIV status disclosure, and community stigma [45–47]. Identifying a specific reason for not starting ART the same day or early should be explored with these participants in order to address this gap in future clients.

### 4.3. Limitations of the Study

This study is the first in the Oromia Region to identify factors associated with AHD, though its findings may not apply to other areas in Ethiopia. It lays a foundation for policymakers to create targeted interventions to prevent AHD and improve health outcomes for PLHIV. Researchers faced challenges with incomplete medical records, leading to the exclusion of missing data on ART follow‐up, baseline viral load tests, HCG results, and mental health status from the analysis. Thus, proper documentation must be prioritised by policy and programme managers.

## 5. Conclusion

The study identified sociodemographic and clinical practice key factors significantly associated with AHD, including a higher risk for males compared to females, individuals showing signs and symptoms of HIV and OIs, severely ill or bedridden patients at ART initiation, those with baseline CD4 test results, and late ART initiation. These findings underscore the need for targeted interventions for ALHIV to prevent and manage AHD through early diagnosis, timely ART initiation and regular screenings. Improved adherence to protocols, prophylaxis provision, and monitoring can enhance treatment outcomes and quality of life for PLHIV in the West Oromia Region.

Further studies are needed to address late health‐seeking behaviours among males to reduce late ART initiation and AHD.

**Nomenclature**
AIDS: Acquired immunodeficiency syndrome
AHD: Advanced HIV disease
ALHIV: Adults living with HIV
BMI: Body mass index
ART: Antiretroviral therapy
AOR: Adjusted odds ratio
CD4 cell: Clusters of differentiation 4
CM: Cryptococcal meningitis
CrAG: Cryptococcal antigen
CPT: Cotrimoxazole prophylaxis therapy
EMRs: Electronic medical records
HB: Haemoglobin
HIV: Human immunodeficiency virus
HCPs: Healthcare providers
IPT: Isoniazid preventive therapy
LMICs: Low‐ and middle‐income countries
MOHSS: Ministry of Health and Social Services
OIs: Opportunistic infections
TB: Tuberculosis
TPT: Tuberculosis preventive therapy
PITC: Provider‐initiated testing and counselling
PLHIV: People living with HIV
UNAIDS: Joint United Nations Programme on HIV/AIDS
UAN: Unique ART number
VCT: Voluntary counselling and testing

## Author Contributions

Geleta Asebe Hinkosa conceptualised the study, collected and analysed data and drafted the manuscript. Sheillah Hlamalani Mboweni contributed to the verification of data analysis. Geleta Asebe Hinkosa and Sheillah Hlamalani Mboweni wrote the manuscript.

## Funding

The study receives no funding.

## Consent

The authors have nothing to report.

## Conflicts of Interest

The authors declare no conflicts of interest.

## Data Availability

The datasets used during the current study will be available on request to the corresponding author.
